# Effect of respiratory muscle training on load sensations in people with chronic tetraplegia: a secondary analysis of a randomised controlled trial

**DOI:** 10.1038/s41393-023-00920-3

**Published:** 2023-08-16

**Authors:** Billy L. Luu, R. H. Chaminda Lewis, Rachel A. McBain, Simon C. Gandevia, Claire L. Boswell-Ruys, Jane E. Butler

**Affiliations:** 1 Spinal Cord Injury Research Centre, Neuroscience Research Australia, Randwick, NSW Australia; 2 University of New South Wales, Sydney, NSW Australia; 3 Prince of Wales Hospital, Randwick, NSW Australia

**Keywords:** Respiration, Neurophysiology

## Abstract

**Study design:**

Secondary analysis of a randomised controlled trial.

**Objectives:**

Our primary study showed that increasing inspiratory muscle strength with training in people with chronic (>1 year) tetraplegia corresponded with reduced sensations of breathlessness when inspiration was loaded. This study investigated whether respiratory muscle training also affected the respiratory sensations for load detection and magnitude perception.

**Setting:**

Independent research institute in Sydney, Australia.

**Methods:**

Thirty-two adults with chronic tetraplegia participated in a 6-week, supervised training protocol. The active group trained the inspiratory muscles through progressive threshold loading. The sham group performed the same protocol with a fixed threshold load (3.6 cmH_2_O). Primary measures were load detection threshold and perceived magnitudes of six suprathreshold loads reported using the modified Borg scale.

**Results:**

Maximal inspiratory pressure (Pi_max_) increased by 32% (95% CI, 18–45) in the active group with no change in the sham group (*p* =  0.51). The training intervention did not affect detection thresholds in the active (*p* =  0.24) or sham (*p* =  0.77) group, with similar overall decreases in Borg rating of 0.83 (95% CI, 0.49–1.17) in active and 0.72 (95% CI, 0.32–1.12) in sham group. Increased inspiratory muscle strength reduced slope magnitude between Borg rating and peak inspiratory pressure (*p* =  0.003), but not when pressure was divided by Pi_max_ to reflect contraction intensity (*p* =  0.92).

**Conclusions:**

Training reduces the sensitivity of load sensations for a given change in pressure but not for a given change in contraction intensity.

## Introduction

Breathing is impaired following cervical spinal cord injury. The loss in respiratory muscle strength leads to reduced lung volumes and capacities and the lungs and chest wall become less compliant [1, 2]. Therefore, our primary study investigated the use of progressive respiratory muscle training (RMT) in restoring some of the loss in inspiratory muscle strength in people with acute or chronic tetraplegia [3] as inspiratory muscle strength is the strongest predictor of respiratory complications [4]. We found that, in people with chronic tetraplegia, a 32% increase in maximal inspiratory pressure (Pi_max_) corresponded to a reduction in respiratory complications, an improvement in quality of life, and a reduction in the severity of breathlessness when inspiration was loaded over multiple breaths. Hence, this secondary analysis was conducted to determine whether RMT also affected the respiratory sensations to detect and perceive an inspiratory load.

Training-induced increases in Pi_max_ of 51% and 60% in healthy able-bodied adults reduce the perceived magnitudes of inspiratory loads [5, 6]. The opposite effect is observed when the inspiratory muscles are temporarily weakened through fatigue [7] or partial paralysis [8]: a given inspiratory load is perceived to be larger. This inverse relationship between perceived magnitude and inspiratory muscle strength suggests that the perceptual response to loading is related to the effort of contraction, a sensation that originates centrally and is proportional to the size of the motor command to the contracting muscles [9]. In the case of RMT, as inspiratory muscle strength increases, the effort required to produce a given inspiratory pressure (force) is reduced as the contraction intensity represents a smaller proportion of the improved Pi_max_. The perceived magnitude of an inspiratory load is indirectly related to added resistance but directly related to peak inspiratory pressure (Pi_peak_) [10, 11]. Accordingly, the direct relationship between the perceived magnitude of an inspiratory load and Pi_peak_ is shifted downwards after inspiratory muscle training [e.g. 5, 6]; although this training-induced shift in load magnitude perception disappears when Pi_peak_ is expressed relative to Pi_max_.

Generalising from findings in healthy able-bodied participants to people with chronic tetraplegia is problematic. Load sensation is normal in people with chronic tetraplegia when perceived magnitudes are plotted against Pi_peak_ divided by Pi_max_ (%Pi_max_) to reflect contraction intensity, but is more sensitive than in healthy able-bodied participants when perceived magnitudes are plotted against absolute Pi_peak_ [12]. It is not clear whether a training-induced improvement in Pi_max_ would cause the relationship between perceived magnitude and Pi_peak_ to become less steep, and therefore normalise with healthy able-bodied participants, or shift downwards as shown in previous inspiratory muscle training studies [5, 6]. Thus, one aim of the present study was to examine the effect of RMT on the perceived magnitudes of six suprathreshold loads in people with chronic tetraplegia. The second aim was to determine whether the training intervention affected their ability to detect an inspiratory load. The means by which a load is detected is not as well understood as how the magnitude of a load is perceived. Unlike load magnitude perception, strengthening [5, 13] or weakening [8] the inspiratory muscles has no effect on load detection threshold in healthy able-bodied participants. However, the detection threshold is 50% higher in people with chronic tetraplegia than in able-bodied participants [12]. Whether a training-induced increase in inspiratory muscle strength can improve impaired load detection has not been investigated.

## Methods

### Definition of terms

Pi_peak_ is the peak inspiratory pressure generated during a loaded or unloaded breath. Pi_max_ is the maximal inspiratory pressure generated against a closed airway at function residual capacity. Pi_peak_ (%Pi_max_) is peak inspiratory pressure expressed as a percentage of Pi_max_ to account for differences in muscle strength between participants; it also reflects the contraction intensity of the inspiratory muscles.

### Study design

Secondary analysis of a double-blind, randomised controlled trial to compare the effect of RMT on the perception of inspiratory loads. The trial was registered at Australian New Zealand Clinical Trials Registry (ACTRN 12612000929808) and conducted at a single site between May 2014 and October 2016.

### Participants

**Fig. 1:**
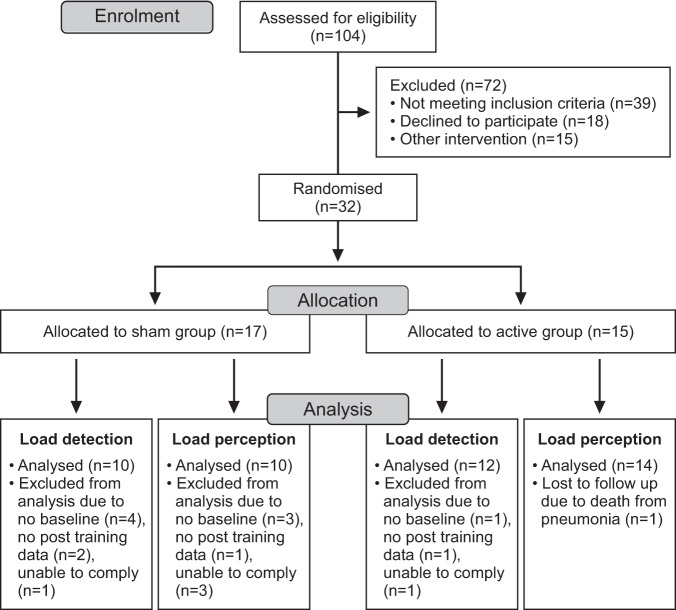
Participant flow diagram. Number (*n*) of participants enrolled and allocated to each group.

Thirty-two participants were recruited from Prince of Wales Hospital and the community in Sydney, Australia (Fig. 1). Eligibility included adults ( ≥18 years) with tetraplegia between neurological levels of C3 and C7, injury-related deficits in respiratory muscle strength, American Spinal Injury Association Impairment Scale classifications of grade A-C, and duration of injury of at least 1 year (see Table 1). People who were mechanically ventilated, pregnant, or diagnosed with coexisting respiratory or neuromuscular disorders or cognitive impairments were excluded.

**Table 1:** Participant characteristics.

	Sham group *n* = 10	Active group *n* = 14
Age, years	54 (11)	54 (9)
Sex, %male	80	100
Height, m	1.76 (0.05)	1.81 (0.04)
Weight, kg	77 (21)	81 (16)
Body-mass index, kg/m^2^	25 (7)	25 (5)
Time since injury, years	24 (16)	26 (11)
Neurological level of Injury, *n*		
C3	1	3
C4	3	3
C5	2	4
C6	3	3
C7	1	1
ASIA Impairment Scale, *n*		
A - motor complete	7	6
B - motor complete	−	8
C - motor incomplete	3	−

*SD* mean, *n* number of participants included in the analyses, *ASIA* American Spinal Injury Association.

### Randomisation and blinding

The primary analysis in Boswell-Ruys et al. [3] used an adaptive random allocation schedule to minimise imbalances between treatment groups. Only people with chronic (>1 year) tetraplegia were included in the present study, but the random allocation sequence still holds due to an initial stratification by time since injury (see [3] for randomisation method). Participants, treating therapists, and assessors were blinded to the allocated treatment group for the trial duration.

### Study intervention

Participants performed a 6-week supervised intervention protocol with an RMT device (Threshold IMT, Philips Respironics, Tangmere, UK); see [3] for details about device modification for active and sham groups, and for inspiratory and expiratory muscle training.

Training sessions were performed twice daily, 5 days a week for 6 weeks. For each session, participants completed three to five sets of 12 breaths for inspiratory and then expiratory muscle training. Each set was separated by two minutes of quiet breathing. Training intensity was initially set at 30% of the participant’s Pi_max_ and 30% of the participant’s maximal expiratory pressure (Pe_max_). Training intensity increased each week by 10%, as calculated from the weekly-measured Pi_max_ and Pe_max_ as strength improved [see ref. 3].

### Outcomes

The primary outcome was the sensory perception of inspiratory loads after RMT. We quantified these sensations in separate load detection and load magnitude perception tasks before and after the intervention. The methods for both tasks have been described previously in detail [see refs. 12, 14]. Briefly, the detection threshold for an inspiratory resistive load was determined using a staircase procedure with a step change in resistance of 0.17 cmH_2_O/l/s up to a maximum of 1.7 cmH_2_O/l/s. An inspiratory load was applied once every few breaths. Participants were prompted to indicate whether the next loaded breath felt different or not when breathing in compared to the current unloaded breath (i.e., baseline). Participants performed two trials: an incrementing and then a decrementing staircase. A single detection threshold was calculated from the average of both trials.

For load magnitude perception, participants rated their perceived efforts of breathing through six non-linear resistive loads using the modified Borg scale [15]. Pressure versus flow characteristics for each load are presented in Luu et al. [12]. Resistance increased sequentially from loads one to six. An inspiratory load was applied once every few breaths. The six loads were presented three times each in random order. Borg ratings for perceived effort were averaged for each load. The changes in Pi_peak_, Pi_peak_ (%Pi_max_), and inspiratory time for each loaded breath relative to the previous unloaded breath were averaged for each of the six loads.

Respiratory measures of mouth pressure (negative for inspiration), flow, and tidal volume were recorded as per Luu et al. [12]. Lung function measures in Table 2 were measured as per Boswell-Ruys et al. [3].

**Table 2:** Lung function and load detection.

	Sham group			Active group			Interaction effect
	Pre	Post	*p* value	Pre	Post	*p* value	*p* value
Lung function measures							
Pi_max_^a^, cmH_2_O	−54 (15)	−56 (17)	0.53	−47 (29)	−62 (29)	<0.001	0.02
Pe_max_^b^, cmH_2_O	35 (13)	35 (14)	0.98	27 (11)	29 (12)	0.37	0.57
Total lung capacity, l	4.9 (1.2)	4.8 (1.1)	0.49	4.9 (1.4)	4.7 (1.5)	0.95	0.64
Inspiratory capacity, l	2.1 (0.5)	2.1 (0.5)	0.98	2.0 (0.8)	1.9 (0.8)	0.60	0.75
Vital capacity, l	2.6 (0.7)	2.6 (0.8)	0.88	2.4 (1.0)	2.3 (1.1)	0.48	0.56
Resting breathing values							
Pi_peak_, cmH_2_O	−0.93 (0.27)	−0.91 (0.19)	0.85	−0.83 (0.26)	−0.85 (0.21)	0.82	0.77
Inspiratory time, s	1.8 (0.4)	1.8 (0.5)	0.48	1.8 (0.6)	1.7 (0.4)	0.29	0.88
Tidal volume, l	0.72 (0.14)	0.81 (0.28)	0.20	0.70 (0.24)	0.67 (0.26)	0.58	0.18
Mean inspiratory flow, l/s	0.42 (0.08)	0.48 (0.11)	0.04^c^	0.39 (0.12)	0.40 (0.10)	0.76	0.16
Detection threshold							
Incrementing, cmH_2_O/l/s	0.83 (0.52)	0.70 (0.55)	0.50	0.81 (0.48)	0.77 (0.64)	0.82	0.73
Decrementing, cmH_2_O/l/s	1.4 (0.3)	1.3 (0.2)	0.20	1.4 (0.2)	1.5 (0.2)	0.65	0.21
Single-point, cmH_2_O/l/s	1.2 (0.4)	1.0 (0.3)	0.26	1.1 (0.3)	1.1 (0.4)	0.78	0.31

Pi_peak_ is negatively increasing. There were no differences in pre-training values between the active and sham groups.

*SD* mean, *Pi*_*max*_ maximal inspiratory pressure, *Pe*_*max*_ maximal expiratory pressure, *Pi*_*peak*_ peak inspiratory pressure.

^a^Indicates performed at functional residual capacity.

^b^Indicates performed at total lung capacity.

^c^Indicates main effect of training intervention (pre vs post) was not significant (*F*_1,22.0_ = 3.5, *p* = 0.08).

### Statistical analysis

Pre-training data from 11 of 32 participants have been presented previously by Luu et al. [12]. Up to ten participants were excluded from the analyses (Fig. 1). For load detection, a mixed linear model fit by restricted maximum likelihood estimation assessed the effects of treatment group (active vs. sham), training intervention (pre vs. post), and their interaction on detection thresholds. Random intercepts were included for participants. For load magnitude perception, the effects of treatment group and training intervention on Borg effort rating, Pi_peak_, or Pi_peak_ (%Pi_max_) were determined in separate mixed linear models with log-transformed added resistance as a mean-centred covariate. The effects of treatment group and training intervention on Borg effort rating were also determined with inspiratory pressure (Pi_peak_ or Pi_peak_ (%Pi_max_)) and inspiratory time as mean-centred covariates instead of log-transformed added resistance. The three-way interaction between each covariate with the treatment group and training intervention, and all lower-order interactions, were included in the mixed linear models with random intercepts for participants. For models with inspiratory time as a covariate, random slopes for inspiratory time were included with an unstructured covariance matrix as participants adopted their own breathing pattern during loading. For lung function and resting breathing measures, separate mixed linear models with random intercepts for participants determined the effects of the treatment group, training intervention, and their interaction on each measure in Table 2. All statistical analyses were performed with IBM SPSS Statistics (v25, IBM Corp., Armonk, NY, USA).

## Results

Table 1 shows the participant characteristics of those included in the analyses.

### Training intervention

The 6-week training protocol increased Pi_max_ by 32% (95% CI, 18 to 45) in the active group but not in the sham group where the increase was 4.5% (95% CI, −9.3 to 18.3), as determined from pairwise comparisons of the estimated marginal means pre and post-training intervention. RMT had no effect on other lung function measures, or on resting breathing values measured immediately prior to the sensory tasks when comparing between the active and sham groups (Table 2).

### Outcomes

**Fig. 2:**
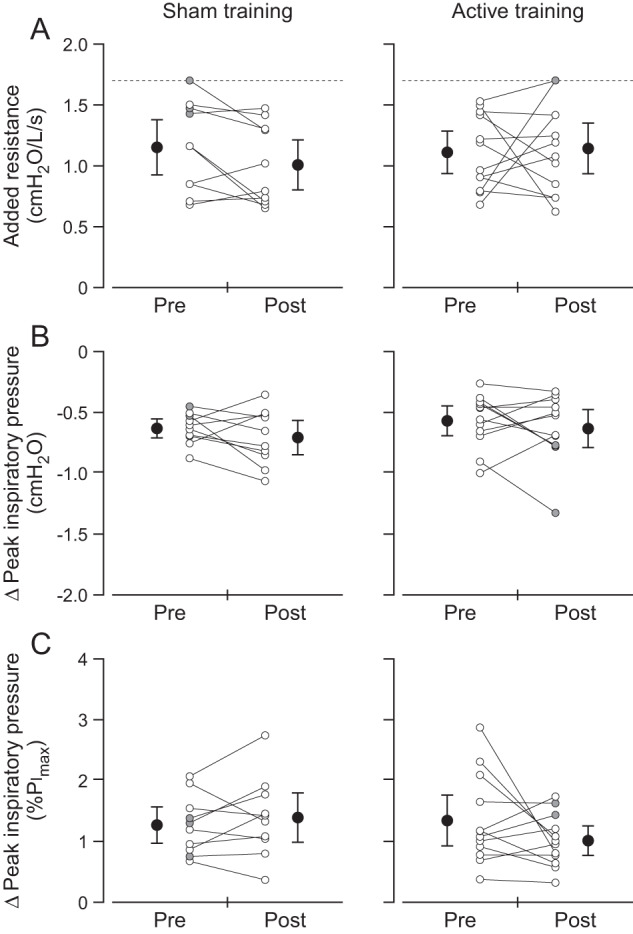
Load detection thresholds. Individual (white circles) and group mean (black circles) data for single-point detection threshold (**A**) and the increase in peak inspiratory pressure at detection threshold (**B**). In (**C**), peak inspiratory pressure is expressed as a percentage of maximal inspiratory pressure (%Pi_max_). Group data are shown as means with 95% confidence intervals. Peak inspiratory pressure is negatively increasing and represents the change (Δ) relative to the previous unloaded breath. There were no significant differences in detection threshold, or peak inspiratory pressure and peak inspiratory pressure expressed as %Pi_max_ at detection threshold, in the sham group (*n* = 10) or active group (*n* = 12) due to the training intervention (pre vs. post). Grey circles show participants who did not detect the loads, and therefore their threshold was taken from the largest load of 1.7 cmH_2_O/l/s (dashed horizontal lines in **A**) in either the incrementing or decrementing trial. A single-point detection threshold of 1.7 cmH_2_O/l/s indicates that a participant did not detect the largest load in the incrementing and decrementing trials.

For the load detection task, some participants did not detect a load when breathing through the largest resistance (see Fig. 2). For these participants, the detection threshold was taken from the largest resistance of 1.7 cmH_2_O/l/s.

RMT had no effect on load detection threshold (Table 2). At detection threshold, there was no main effect of training intervention on Pi_peak_ (F_1,20_  =  1.8, *p*  =  0.19) or Pi_peak_ (%Pi_max_) (F_1,20_  =  0.57, *p* =  0.46) as shown in Fig. 2B, C, respectively; nor was there an interaction effect with treatment group for Pi_peak_ (*F*_1,20_  =  0.01, *p* =  0.92) or Pi_peak_ (%Pi_max_) (*F*_1,20_  =  2.7, *p* =  0.12).

**Fig. 3:**
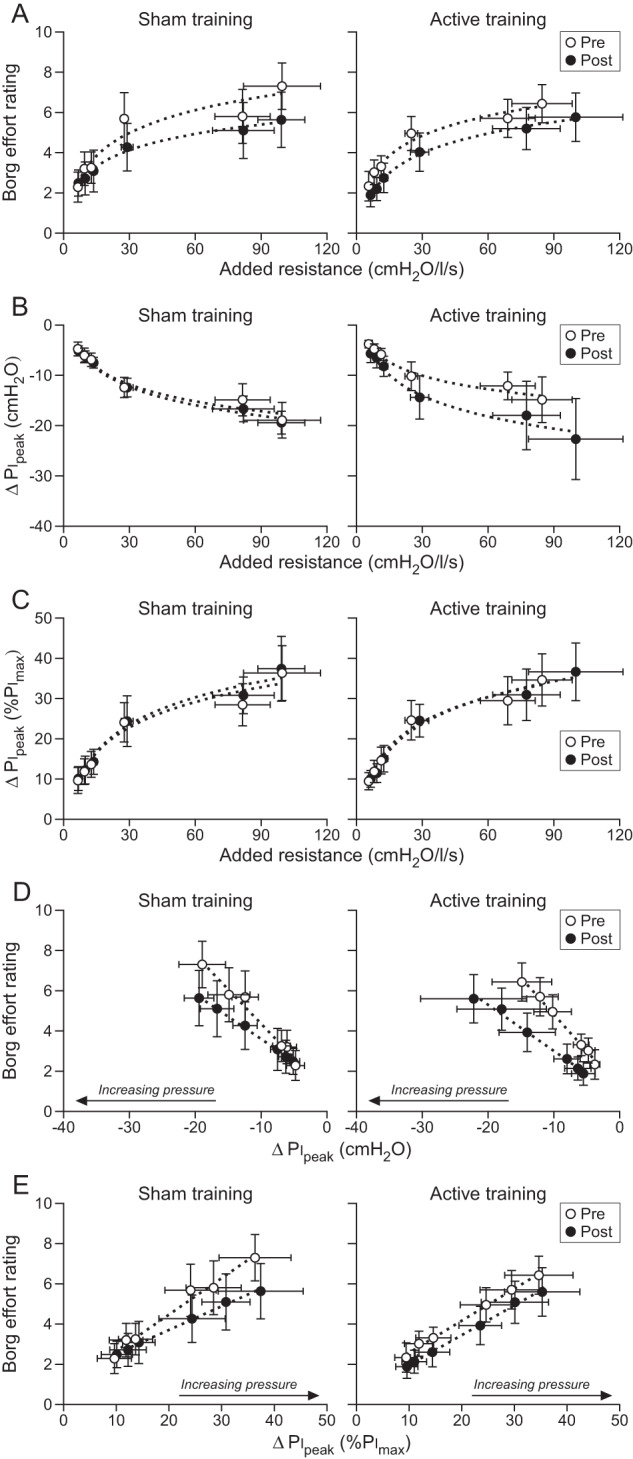
Load magnitude perception. Group mean data with 95% confidence intervals for the sham group (*n* = 10; left panels) and active group (*n* = 14; right panels). Dotted trendlines represent linear or logarithmic best fits of the mean data for each group and not of the coefficient estimates from the mixed model analyses (see Table 4). Peak inspiratory pressure (Pi_peak_) is negatively increasing and represents the change (Δ) relative to the previous unloaded breath. **A** Borg effort rating increased with added resistance in both treatment groups. The decrease in mean Borg rating due to the training intervention (pre vs. post) was similar between the sham and active groups when evaluated at the mean log-transformed added resistance (*p* =  0.68). **B** Pi_peak_ increased with added resistance. Mean Pi_peak_ was greater post training in the active group (*p* <  0.001) but there was no effect of training intervention on mean Pi_peak_ in the sham group (*p* =  0.61). **C** Pi_peak_ divided by maximal inspiratory pressure (Pi_max_) to reflect contraction intensity. **D**. The increase in Borg effort rating with increasing negative Pi_peak_ was less in the active group after the training intervention (*p* =  0.003) but there was no change in the sham group (*p* =  0.13). **E** There was no effect of training intervention on slope magnitude for the relationship between Borg effort rating and contraction intensity (%Pi_max_) in both the active (*p* =  0.92) and sham (*p* =  0.61) groups.

In the load magnitude perception task, Borg effort rating increased with added resistance in both treatment groups (Fig. 3A and Table 3). Mean Borg ratings were 4.5 (95% CI, 3.7–5.4) for the sham group and 4.4 (95% CI, 3.7–5.2) for the active group when evaluated at the mean log-transformed resistance. Following the training intervention, Borg ratings decreased by 0.72 (95% CI, 0.32–1.13) in the sham group and 0.83 (95% CI, 0.48–1.19) in the active group, a similar decrease between treatment groups (*F*_1,253.0_ =  0.16, *p* =  0.69). The slopes between Borg effort rating and log-transformed resistance were similar between treatment groups after the training intervention (Table 4).

**Table 3:** Load magnitude perception.

	Sham group						Active group					
Load	Borg	∆Pi_peak_	∆Pi_peak_	∆T_I_	∆V_T_	∆V̇_I_	Borg	∆Pi_peak_	∆Pi_peak_	∆T_I_	∆V_T_	∆V̇_I_
		(cmH_2_O)	(%Pi_max_)	(s)	(l)	(l/s)		(cmH_2_O)	(%Pi_max_)	(s)	(l)	(l/s)
Pre-training												
1	2.3 (1.2)	−4.8 (2.2)	10 (5)	0.27 (0.28)	0.06 (0.11)	−0.05 (0.09)	2.3 (1.4)	−3.8 (1.5)	9.4 (4.1)	0.36 (0.28)	0.05 (0.10)	−0.06 (0.05)
2	3.2 (1.3)	−6.1 (2.4)	12 (5)	0.38 (0.31)	0.10 (0.10)	−0.05 (0.10)	3.0 (1.2)	−4.8 (1.9)	12 (5)	0.49 (0.36)	0.06 (0.07)	−0.08 (0.05)
3	3.3 (1.3)	−6.9 (2.2)	14 (5)	0.50 (0.29)	0.01 (0.13)	−0.13 (0.10)	3.3 (1.0)	−5.9 (2.3)	15 (6)	0.60 (0.34)	−0.01 (0.10)	−0.14 (0.07)
4	5.7 (2.1)	−12 (3)	24 (8)	1.1 (0.6)	−0.13 (0.10)	−0.28 (0.09)	5.0 (1.6)	−10 (5)	25 (9)	0.97 (0.63)	−0.20 (0.24)	−0.26 (0.08)
5	5.8 (2.2)	−15 (5)	29 (8)	1.2 (0.6)	−0.20 (0.16)	−0.31(0.14)	5.7 (1.8)	−12 (5)	30 (11)	1.1 (1.0)	−0.35 (0.24)	−0.32 (0.08)
6	7.3 (1.9)	−19 (6)	36 (11)	1.2 (0.8)	−0.49 (0.13)	−0.41 (0.09)	6.4 (1.8)	−15 (9)	35 (12)	1.5 (1.7)	−0.47 (0.26)	−0.38 (0.09)
Post-training												
1	2.5 (1.0)	−5.1 (1.6)	10 (5)	0.44 (0.36)	0.14 (0.26)	−0.06 (0.09)	1.9 (1.1)	−5.5 (3.4)	10 (4)	0.44 (0.44)	0.11 (0.18)	−0.04 (0.08)
2	2.7 (1.3)	−6.2 (1.7)	12 (6)	0.44 (0.27)	0.11 (0.16)	−0.07 (0.08)	2.1 (1.1)	−6.4 (3.8)	11 (4)	0.49 (0.54)	0.10 (0.17)	−0.05 (0.06)
3	3.1 (1.7)	−7.5 (1.7)	14 (5)	0.65 (0.33)	0.10 (0.22)	−0.13 (0.09)	2.6 (1.4)	−8.0 (3.8)	14 (6)	0.82 (0.71)	0.10 (0.16)	−0.10 (0.06)
4	4.3 (1.9)	−12 (3)	24 (10)	1.3 (1.3)	−0.09 (0.35)	−0.29 (0.10)	3.9 (1.8)	−14 (8)	24 (8)	1.1 (0.9)	−0.10 (0.16)	−0.24 (0.08)
5	5.1 (2.3)	−17 (4)	31 (7)	1.5 (1.6)	−0.17 (0.29)	−0.34 (0.11)	5.1 (2.0)	−18 (13)	30 (12)	1.3 (1.0)	−0.21 (0.21)	−0.31 (0.07)
6	5.6 (2.2)	−19 (4)	37 (13)	2.1 (3.3)	−0.42 (0.34)	−0.43 (0.09)	5.7 (2.3)	−22 (15)	36 (14)	1.8 (1.5)	−0.40 (0.21)	−0.39 (0.08)

A negative change (∆) in Pi_peak_ represents a greater negative pressure whereas a negative ∆V_T_ or ∆V̇_I_ represents a smaller value relative to the previous unloaded breath. Post-training data for V_T_ and V̇_I_ from one participant in the active group were omitted from the means due to poor flow recordings.

*SD* mean, *Pi*_*peak*_ peak inspiratory pressure, *T*_*I*_ inspiratory time, *V*_*T*_ tidal volume, *V̇*_*I*_ mean inspiratory flow, *Pi*_*max*_ maximal inspiratory pressure.

**Table 4:** Coefficients of mixed linear models.

	Sham group			Active group			Interaction effect
	Pre	Post	*p* value	Pre	Post	*p* value	*p* value
Slope of Borg vs. log-resistance	3.6 (0.3)	2.7 (0.3)	0.03^a^	3.3 (0.3)	3.3 (0.3)	0.98	0.10
Slope of Pi_peak_ vs. log-resistance	−11.5 (1.2)	−12.0 (1.2)	0.73	−9.2 (1.0)	−15.1 (1.0)	<0.001	0.02
Slope of Pi_peak_ (%Pi_max_) vs. log-resistance	21.1 (1.6)	22.7 (1.6)	0.48	21.0 (1.3)	22.6 (1.3)	0.22	0.80
Slope of Borg vs. Pi_peak_	−0.17 (0.03)	−0.23 (0.03)	0.13	−0.21 (0.03)	−0.13 (0.02)	0.005	0.004
Slope of Borg vs. T_I_	1.6 (0.5)	−0.59 (0.47)	<0.001	1.1 (0.4)	0.53 (0.42)	0.09	<0.001
Slope of Borg vs. Pi_peak_ (%Pi_max_)	0.11 (0.02)	0.10 (0.02)	0.55	0.11 (0.01)	0.11 (0.01)	0.91	0.79
Slope of Borg vs. T_I_	1.1 (0.5)	−0.70 (0.38)	<0.001	0.79 (0.39)	−0.16 (0.33)	0.001	0.04

Pi_peak_ is negatively increasing. Each row of data in rows 1-3 are from separate mixed linear models; data in rows 4 and 5 are from the same mixed linear model; data in rows 6 and 7 are from the same mixed linear model.

*SE* mean, *Pi*_*peak*_ peak inspiratory pressure, *Pi*_*max*_ maximal inspiratory pressure, *T*_*I*_ inspiratory time.

^a^Indicates main effect of training intervention (pre vs post) was not significant (*F*_1,252.4_ = 2.6, *p* = 0.11).

For load magnitude perception, mean change in Pi_peak_ increased by 3.3 cmH_2_O (95% CI, 2.0–4.6) in the active group after training, which was 2.9 cmH_2_O (95% CI, 0.9–4.9) more than the sham group who showed no change in Pi_peak_ (*p* =  0.61). This corresponded with an increase in slope between Pi_peak_ and log-transformed resistance in the active group, but not sham group, after the training intervention (Fig. 3B and Table 4). However, when mean Pi_peak_ was divided by Pi_max_ (Fig. 3C), there was no main effect of training intervention on pressures of 20%Pi_max_ (95% CI, 16–24) in the sham group and 22%Pi_max_ (95% CI, 18–25) in the active group (*F*_1,252.7_  =  0.18, *p* =  0.67). Nor was the change in pressure after the training intervention different between treatment groups (*F*_1,252.7_ =  1.4, *p* =  0.25). The training intervention did not affect the slope between Pi_peak_ (%Pi_max_) and log-transformed resistance (Table 4).

As shown in Fig. 3D, Borg effort rating increased with negative Pi_peak_ (F_1,271.2_ =  136.6, *p* <  0.001). RMT decreased the magnitude of the slope between Borg rating and Pi_peak_ in the active group, a decrease of 0.15 Borg/cmH_2_O (95% CI, 0.05–0.25) more than the sham group who showed no change in slope magnitude (Table 4). The positive relationships between Borg rating and inspiratory time were similar between treatment groups prior to training (*p* =  0.50). The training intervention changed the relationship between Borg rating and inspiratory time (*F*_1,228.4_  =  32.7, *p* <  0.001), but this depended on the treatment group as the slope became negative in the sham group, whereas there was no significant change in the active group (Table 4). Mean Borg rating decreased by a similar amount between treatment groups after the training intervention (*F*_1,254.9_ =  1.7, *p* =  0.20), with decreases of 1.1 (95% CI, 0.6–1.5) in the sham group and 1.5 (95% CI, 1.1–1.9) in the active group.

Dividing Pi_peak_ by Pi_max_ (Fig. 3E) negated the training effect on the relationship between Borg effort rating and Pi_peak_ as there was no difference in the change in slopes between treatment groups (Table 4). With Pi_peak_ (%Pi_max_) as a covariate, the relationship between Borg rating and inspiratory time changed from positive to negative for both active and sham groups post training (Table 4); however, a difference in the change in slope magnitude between treatment groups persisted with a 0.89 Borg/s (95% CI, 0.03–1.75) greater change in the sham group than active group. Mean Borg rating decreased by a similar amount between treatment groups after the training intervention (*F*_1,255.7_  =  0.07, *p* =  0.79), with decreases of 1.0 (95% CI, 0.6–1.5) in the sham group and 0.9 (95% CI, 0.6–1.3) in the active group.

## Discussion

RMT increased Pi_max_ by 32% in people with chronic tetraplegia. This improvement in inspiratory muscle strength affected some aspects of load magnitude perception but had no effect on load detection threshold. For load magnitude perception, there was an overall decrease in Borg effort ratings after the 6-week intervention that was not related to the increase in Pi_max_. However, there was a training-related increase in Pi_peak_ during loaded breaths, which decreased the slope of the relationship between Borg rating and Pi_peak_. That is, for a given change in Pi_peak_, the inspiratory loads were perceived to be less effortful when inspiratory muscle strength improved. The higher Pi_peak_ offset the increase in Pi_max_ so that contraction intensities (%Pi_max_) for the six resistive loads remained the same despite improvements in inspiratory muscle strength. Consequently, the perceptual response to loading did not change after RMT when Borg rating was plotted against Pi_peak_ (%Pi_max_).

Our expectation that improvements in Pi_max_ would reduce the relative contraction intensities of the inspiratory muscles, leading to a given inspiratory load being perceived as less effortful, did not eventuate. Borg ratings decreased after the training intervention, but the active and sham groups had similar changes. This suggests a “learning” effect despite the 6-weeks between test sessions. Participants had no experience breathing with an added resistive load and may have been more comfortable with the inspiratory loading setup during the second test session. In our previous study [14], a similar decrease in Borg ratings was observed in healthy able-bodied participants when the load magnitude perception task was repeated about a week apart with no other intervention. Eastwood et al. [16] suggested that changes in breathing patterns could explain the learning effect observed for progressive threshold loading where maximal threshold pressure increased and rate of perceived exertion decreased over successive days when there was no change in Pi_max_. In the present study, there was no difference in resting breathing values between test sessions (Table 2), but participants may have slightly altered inspiratory times for loaded breaths after their first session of the load magnitude perception task (Table 3), as will be discussed later. Alternatively, Boswell-Ruys et al. [3] reported in the primary study that the resistance of the sham training device was not negligible, requiring a threshold pressure of 3.6 cmH_2_O. It is possible then that the stimulus generated by the sham device was sufficient to induce a training effect on Borg ratings without increasing inspiratory muscle strength. The learning-effect explanation seems more plausible, which would have significant implications for previous training studies in healthy able-bodied participants as they do not include a control (sham) group for comparison [e.g. 5, 6]. Pi_max_ increased by 51% and 60% in those previous studies, almost twice as large as the 32% improvement obtained in this study, so the likelihood of a training-related decrease in load ratings as reported in [5, 6] is greater if a training effect was present. Since the contribution from a learning effect on load ratings cannot be determined in those previous studies, their findings should be interpreted with caution as the training-related decreases in perceived magnitudes may be overstated.

RMT did not directly lower Borg ratings. However, the 32% improvement in inspiratory muscle strength decreased the slope of the relationship between Borg rating and Pi_peak_ by 38% (Table 4) so the change in perceived magnitude of an inspiratory load was less sensitive to a given change in absolute Pi_peak_. This decrease in slope magnitude was achieved primarily through an increase in Pi_peak_, which was unexpected as the same set of resistive loads was used for both test sessions. In healthy able-bodied participants, Pi_peak_ did not increase after inspiratory muscle training for loads ranging from 3.5 to 50.6 cmH_2_O/l/s when inspiratory flow was unconstrained [6]. For people with chronic tetraplegia, it is not clear why there was a consistent increase in Pi_peak_ in the active group after training (Fig. 3B). It appears that participants were unaware that the force of contraction generated by the strengthened inspiratory muscles was more than adequate to overcome the added resistance to breathing. The loss or impairment of afferent inputs from muscles acting on the chest wall is probably important here as the increase in Pi_peak_ kept contraction intensity (central drive) the same after training, as indicated by the lack of a training-related difference in slopes between Pi_peak_ (%Pi_max_) and log-transformed resistance, a behaviour more consistent with an open-loop control system. As a result, when Borg rating was plotted against Pi_peak_ (%Pi_max_) to represent contraction intensity (Fig. 3D), the training effect on slope magnitude disappeared. That is, for a given change in contraction intensity, the inspiratory loads were perceived to be just as effortful irrespective of the absolute Pi_max_. A constant relationship between load ratings and contraction intensity was also observed in healthy able-bodied participants after training [5, 6]; although, constancy was achieved via different means to those with chronic tetraplegia. Here, contraction intensities were maintained as slope magnitudes were reduced whereas in healthy able-bodied participants load ratings and contraction intensities both decreased, shifting the relationship between load rating and Pi_peak_ to the left. Regardless, these findings support the suggestion by Luu et al. [12] that load magnitude perception in chronic tetraplegia reflects the contraction intensity of the inspiratory muscles and not the absolute inspiratory muscle force, and as in healthy able-bodied participants [5, 6], is not affected by training-induced changes in Pi_max_.

As mentioned earlier, participants seemed to have altered their breathing pattern during loaded breaths in the second session of the load magnitude perception task. The direct relationship between the perceived magnitude of an inspiratory load and inspiratory time in the first test session (Table 4) replicates what has been reported previously [e.g. 11, 12, 17]. However, after the 6-week intervention, the positive relationship between Borg rating and inspiratory time was inverted in the sham group when Pi_peak_ was used as the covariate in the mixed linear model; no change in the relationship was observed in the active group, perhaps due to the increased Pi_peak_ after training. When Pi_peak_ was expressed as a percentage of Pi_max_, the relationship between Borg rating and inspiratory time changed from positive to negative in both the active and sham groups. Whether this behaviour is normal or limited to chronic tetraplegia is unknown. Of the previous studies that have repeated a load magnitude perception task in the same participants, two did not investigate the relationship between load ratings and inspiratory time [5, 14] and the other assumed a positive relationship and divided load ratings by a fixed power of inspiratory time [6]. Inspiratory time was identified as an important component in the learning effect observed for progressive threshold loading [16]. For that task, inspiratory times decreased following successive tests, presumably to increase the recovery time of the inspiratory muscles and prolong endurance. Here, the inverse relationship in the second test session indicates longer inspiratory times were associated with lower Borg ratings. While inspiratory times appeared to increase during the second session, there was no accompanying decrease in inspiratory flow, and hence pressure, that would be required to reduce load sensations. Further investigations are needed to understand the relationship between load ratings and inspiratory time as our findings suggest that a positive relationship should not be assumed when testing non-naïve participants or conducting repeated sessions on the same participants.

Increased Pi_max_ did not lower load detection thresholds. Nor did repeating the load detection task in the sham group after the training intervention, although the underestimation of detection thresholds in the sham group prior to training may have masked any learning effect (Fig. 2A). These results reproduce those found in healthy able-bodied participants [5, 13, 14] and indicate that load detection is not dependent on inspiratory muscle strength.

Not all participants completed the load detection and load magnitude perception tasks (Fig. 1), and this limitation resulted in an unevenly distributed study population for level of injury and motor completeness (Table 1). However, neither factor alone indicates the level of inspiratory muscle impairment as Pi_max_ was similar between the sham and active groups prior to RMT (Table 2). Moreover, our findings showed that, for respiratory sensations, contraction intensity was the determining factor in a participant’s perceived effort of an inspiratory load. As this was a secondary analysis of a randomised controlled trial, it is possible that the present study was not adequately powered to compare differences between the sham and active groups for our non-significant findings.

In conclusion, our findings present a dichotomy for recommendation of RMT as part of rehabilitation for people with chronic tetraplegia. On the one hand, improved inspiratory muscle strength reduced the slope of the relationship between Borg rating and absolute Pi_peak_, which suggests RMT has the potential to lessen the increase in perceived effort when the resistance to breathing increases. For example, this would occur during respiratory distress from pneumonia or exercise, exacerbations of asthma or airway disease, or from weight gain. On the other hand, RMT did not produce an overall decrease in Borg effort ratings across the loads, which suggests a limited benefit in prescribing RMT solely to reduce perceived effort of breathing at rest. However, this should not be considered a contraindication for RMT as increasing inspiratory muscle strength also reduces respiratory complications and improves quality of life [3]. Selection of a suitable training load that will increase muscle strength while minimising breathing discomfort is therefore important to optimise recovery. The comparable relationships between Borg effort rating and contraction intensity for people with chronic tetraplegia and able-bodied controls [12] suggest training protocols that are well tolerated by able-bodied participants will equally be well tolerated by people with chronic tetraplegia, as long as training protocols are based on changes in contraction intensity relative to maximum rather than the physical properties of the load.

## Supplementary information

Reproducibility checklist

## Data Availability

Data are available from the corresponding author upon reasonable request.
